# Combining Problem-Based Learning Methods With the WeChat Platform in Teaching Ophthalmology: Randomized Controlled Trial

**DOI:** 10.2196/65279

**Published:** 2026-01-05

**Authors:** Fang Fang, Bing Bu, Wenmin Jiang

**Affiliations:** 1 Department of Ophthalmology in the Second Xiangya Hospital, Central South University, 139 Renmin Road, Changsha, 410011, China, 86 13574897769

**Keywords:** problem-based learning, WeChat, China, ophthalmology, undergraduate, medical students

## Abstract

**Background:**

Ophthalmology poses distinct learning challenges for medical students due to the complex anatomy of the eye and the requirement of essential hands-on skills. Problem-based learning (PBL), a student-centered approach, fosters clinical reasoning and self-directed learning. To address the time and logistical constraints of traditional teaching methods, this study implemented a WeChat-based PBL model that leveraged the platform’s efficiency and interactivity to enhance student engagement and skill acquisition in ophthalmology.

**Objective:**

This study aimed to evaluate the effectiveness of WeChat-based PBL in ophthalmology education, focusing on its impact on students’ self-perception of learning and clinical skills compared to traditional teaching methods.

**Methods:**

This study involved 108 undergraduate students who successfully passed the Chinese National Entrance Examination. Among them, 54 (50%) were randomly selected to participate in the WeChat-based PBL, while the other 54 (50%) received traditional teaching. Students were placed into 6 groups (18 students for each group) using a random number table, and the new teaching methods were tested outside their regular class time. Three groups were randomly selected to receive PBL using WeChat as the platform, while the remaining 3 groups received conventional teaching.

**Results:**

Our analysis indicated that although students in the WeChat-based PBL group scored marginally lower in memorization compared to their peers in the traditional teaching group (traditional group: mean 37.6, SD 2.8; WeChat group: mean 32.0, SD 4.1; *P*=.006; n=54), they exhibited markedly superior levels of understanding (traditional group: mean 24.1, SD 1.8; WeChat group: mean 28.0, SD 1.3; *P*=.007; n=54) and knowledge application (traditional group: mean 24.3, SD 1.9; WeChat group: mean 27.6, SD 1.3; *P*=.008; n=54). This suggests that the WeChat-based PBL method promotes deeper engagement, enabling students to better comprehend essential concepts, even with a diminished emphasis on rote learning. Additionally, students in the WeChat group reported increased collaboration (traditional group: mean 3.8889, SD 0.8393; WeChat group: mean 1.7222, SD 0.5961; *P*<.001); motivation (traditional group: mean 3.5471, SD 0.7915; WeChat group: mean 1.8333, SD 0.5746; *P*=.004); knowledge acquisition (traditional group: mean 3.6667, SD 0.7770; WeChat group: mean 1.8704, SD 0.7017; *P*<.001); self-learning ability (traditional group: mean 3.5741, SD 0.7673; WeChat group: mean 1.8519, SD 0.4917; *P*<.001); clinical reasoning (traditional group: mean 2.9444, SD 0.8777; WeChat group: mean 1.9630, SD 0.6132; *P*=.01); and problem-solving skills (traditional group: mean 3.2037, SD 0.6553; WeChat group: mean 1.8519, SD 0.5287; *P*=.001) than the students in the traditional group.

**Conclusions:**

Integrating PBL methods with WeChat has been shown to improve ophthalmic education outcomes compared to traditional teaching, suggesting that this method may offer a superior alternative to conventional teaching.

## Introduction

Medical education increasingly incorporates innovative strategies in undergraduate training to better prepare students for the challenges of clinical practice. Key strategies such as problem-based learning (PBL) [1,2], case-based learning [3], team-based learning [4], flipped classroom–based learning [5,6], and competency-based medical education [7] have been emphasized to bridge the gap between theoretical knowledge and clinical skills. These strategies are particularly relevant in fields such as ophthalmology, which represents a distinct and highly specialized discipline within clinical medicine [8]. The intricate anatomy of the eye and the nonintuitive nature of its structure necessitate the use of specialized equipment and tests for accurate diagnosis and management [9]. These factors contribute to the considerable hurdles medical students encounter while attempting to master this subject. Therefore, effective teaching methodologies are vital for adequately preparing students for clinical practice and developing their competencies as future clinicians [10].

The conventional teacher-centered educational model often relies on the passive transmission of knowledge from instructors to students. While efficient for delivering core knowledge, especially in large classes common in China, this method can limit students’ engagement and active learning [11]. As a result, students may struggle to operate ophthalmic instruments or perform fundus examinations, thereby diminishing their confidence in applying ophthalmology skills. Moreover, although textbooks provide detailed descriptions of the clinical manifestations and signs of ophthalmic diseases, students frequently encounter challenges when attempting to apply this theoretical knowledge to real-world clinical scenarios [12].

PBL, which inherently starts with a clinically based problem as its name, is an active learning strategy that fosters student-centered engagement [13]. Evidence shows that PBL enhances learning motivation, promotes self-guided learning, and improves the integration of theoretical knowledge with clinical practice [14,15]. Students exposed to PBL report greater satisfaction and participation compared with those in traditional learning settings [16,17]. In today’s digital era, college students are adept at using the internet and mobile networks to access information, helping to overcome the temporal and spatial limitations of traditional PBL methods. With the advancement of mobile networks, WeChat has become a popular social networking platform in China [18]. It supports multiple modes of interaction—including texting, voice messages, images, and multimedia—similar to WhatsApp in Western countries [19,20]. Moreover, WeChat is widely used for disseminating knowledge to medical students due to its convenience and the extensive availability of information [21,22].

This study addresses the challenges faced in conventional ophthalmology education, where passive learning hinders the development of practical skills and confidence among undergraduate medical students. It aims to evaluate the effectiveness and acceptability of a WeChat-based PBL approach in enhancing students’ self-perceptions of learning, initiative, organization, clinical skills, and knowledge mastery. The central research question guiding this inquiry is: How does the integration of WeChat in PBL influence the learning outcomes of medical students in ophthalmology? This randomized controlled trial will involve the comparison of student outcomes between those engaged in traditional teaching methods and those using the WeChat platform for online PBL, allowing for a robust assessment of the intervention’s effectiveness. We hypothesize that the use of WeChat for online PBL will significantly improve student engagement and competency in ophthalmology. The findings of this study will provide valuable insights for medical educators, curriculum developers, and policymakers seeking to improve medical education methodologies in China and beyond.

## Methods

### Ethical Considerations

This study was conducted in accordance with the Declaration of Helsinki and received approval from the institutional review board and ethics committee of the Second Xiangya Hospital (Z0005-01). In accordance with institutional guidelines for educational research, the intervention was classified as minimal risk and considered part of the routine evaluation of teaching quality. All participants were informed of the study’s purpose, procedures, and the planned use of the collected data, and all provided written informed consent before participation. Participant anonymity was strictly protected, with all data deidentified and analyzed only in aggregated form. No study procedures posed risks beyond those ordinarily encountered in standard educational activities.

### Study Participants

A total of 108 fifth-year undergraduate students from Xiangya Medical College of Central South University, all of whom had passed the Chinese National Entrance Examination, participated in the study. The cohort consisted of 42 (38.9%) male and 66 (61.1%) female participants, aged 20 to 22 years. Participants were randomly assigned into 6 groups of 18 students each using Microsoft Excel to generate random numbers to ensure unbiased distribution. In this study, students were informed of their group assignments due to the nature of the teaching intervention, but evaluators responsible for assessing the examination results were blinded to group allocation to minimize assessment bias. The new teaching methods were implemented outside their regular class schedules, primarily during evenings and weekends. This approach presented unique challenges, including an increased workload for both students and instructors, as it required students to allocate additional time beyond their standard academic hours to participate in the study. Additionally, coordinating schedules among all faculty members and students involved posed challenges, which required careful planning and communication to ensure participation without conflicting with existing institutional educational policies. Both intervention and control groups received instruction in dedicated classrooms at the hospital, which facilitated a conducive learning environment (Table 1).

**Table 1.** Timeline of the WeChat-based problem-based learning activities.

Week	Activity	Duration (h)	Learning objectives
1	The instructor posts guiding questions and students form study groups	2	Students are randomly assigned to study groups of 10 and establish WeChat groups, through which group members communicate about the study content
2	Explore contributing factors	4	Identify and explain major factors and search for medical literature and books on your own to find the answers to the guiding questions provided by the instructor
3	Apply concepts to scenarios	4	Apply learned concepts to hypothetical scenarios and communicate with group members and the instructor through the WeChat group
4	Solve the guiding problems and present the key points	5	Students create relevant course Microsoft PowerPoint presentations according to the assigned groups, which cover all the answers to the instructor’s guiding questions and the knowledge points obtained from self-study of the teaching content of this course
5	Evaluate proposed solutions	4	Evaluate the effectiveness of various solutions by presenting them to each other during the formal class

### Research Methods

We selected primary angle-closure glaucoma (PACG) as the topic for applying the WeChat-based PBL approach in this study, as the diagnosis and treatment of PACG represent crucial skills that students must acquire. The textbook used for this study was *Ophthalmology*, 9th edition, published by the People’s Medical Publishing House [23].

### WeChat-Based PBL

#### Establishment of Learning Groups

Three groups (18 students for each group) were randomly selected to take part in the WeChat-based PBL program. In each group, 1 volunteer self-selected by students was responsible for recording each student’s speech and the outcomes of the panel discussions.

#### Problem Introduction Phase

In alignment with the national syllabus and the ophthalmology textbook, 3 representative clinical cases were selected from the 2022-2023 glaucoma service database of the Second Xiangya Hospital. Each vignette depicted a distinct stage or mechanism of PACG (including asymptomatic primary angle closure, acute angle closure attack, and plateau-iris configuration) covering the essential diagnostic and therapeutic spectrum required for undergraduate training. To guide case selection and design, chapter-level learning objectives were first extracted, encompassing (1) anterior-chamber anatomy and angle structures, (2) the pathophysiology of pupillary-block versus nonpupillary-block mechanisms, (3) biometric risk factors, (4) gonioscopic and imaging diagnosis, (5) laser and surgical indications, and (6) emergency management of acute attacks. Each objective was then translated into a practical key decision point (eg, interpreting a gonioscopy record, determining when laser peripheral iridotomy is indicated, or choosing between phacoemulsification and trabeculectomy). All selected cases, provided by the instructors, were distributed to students 1 week prior to the lecture to facilitate preparatory discussion and independent learning.

#### Preclass Guidance Phase

Instructors provided curated readings including key sections drawn from the textbook, supplemented with selected review articles, clinical guidelines (eg, *Asia-Pacific Glaucoma Guidelines, 3rd Edition*), and representative case reports from the hospital’s glaucoma database. Additionally, each vignette was accompanied by inquiry-based questions that directed students to identify risk factors, compare pathogenic mechanisms, and determine appropriate management strategies, encouraging active and evidence-based learning. These questions could direct students’ literature searches toward key concepts and clinical decision-making points related to PACG. Each group participating in the WeChat-based PBL established its own discussion team on the WeChat platform, fostering an environment of active participation. To encourage full student engagement, instructors emphasized the importance of collaboration and implemented a system that recognized contributions, motivating students to prepare thoroughly before classes. Through this platform, instructors and students engaged in interactive discussions on the provided cases, sharing their diagnostic reasoning, proposing treatment plans, and exploring the latest global developments in disease management. For example, instructors could prompt a discussion by asking specific diagnostic questions, thereby guiding students to reflect critically. During class sessions, instructors monitored and reviewed the discussions and the online outputs, often projecting key discussion points or summaries from WeChat onto a shared screen for collective evaluation and real-time feedback.

#### Discussion and Summarization Phase

Each WeChat-based PBL session lasted 2 hours and was supported by the platform’s features for sharing text, images, voice recordings, videos, and documents, which enabled immediate and efficient communication. All instructors followed a structured facilitator’s guide, identical across groups, and distributed 1 week before the session. The guide provided a minute-by-minute timetable covering the entire 2-hour process (10 min recap, 45 min group presentation, 30 min cross-groups challenge, 25 min instructor synthesis, and 10 min reflection), ensuring balanced pacing and consistent coverage across groups.

At the beginning of each session, a representative student from each group presented their understanding of the assigned disease and summarized unresolved problems identified during preclass discussions. This was followed by an open, interactive discussion in which students from other groups exchanged perspectives and clinical reasoning. To maintain alignment with curricular standards, the instructor’s guide specified 5 “must-cover” learning objectives mapped to the national ophthalmology syllabus (eg, explain the mechanism of pupillary block, justify the timing of laser iridotomy, and interpret ultrasound biomicroscopy images). It also included prompt questions addressing common misconceptions identified during the pilot phase, and a 1-slide “take-home” summary that instructors were required to display at the end of the session.

After the session, instructors completed a structured checklist to document which objectives had been achieved. They were instructed to intervene only when discussion drifted more than 2 minutes off task or when critical misconceptions arose; otherwise, they guided students by inquiry-based questioning to ensure that all checklist objectives were discussed. The structured facilitation process, combined with WeChat’s interactive features, fostered active participation, peer collaboration, and efficient knowledge integration, greatly enhanced the PBL learning experience.

### Traditional Teaching Group

**Figure 1.**
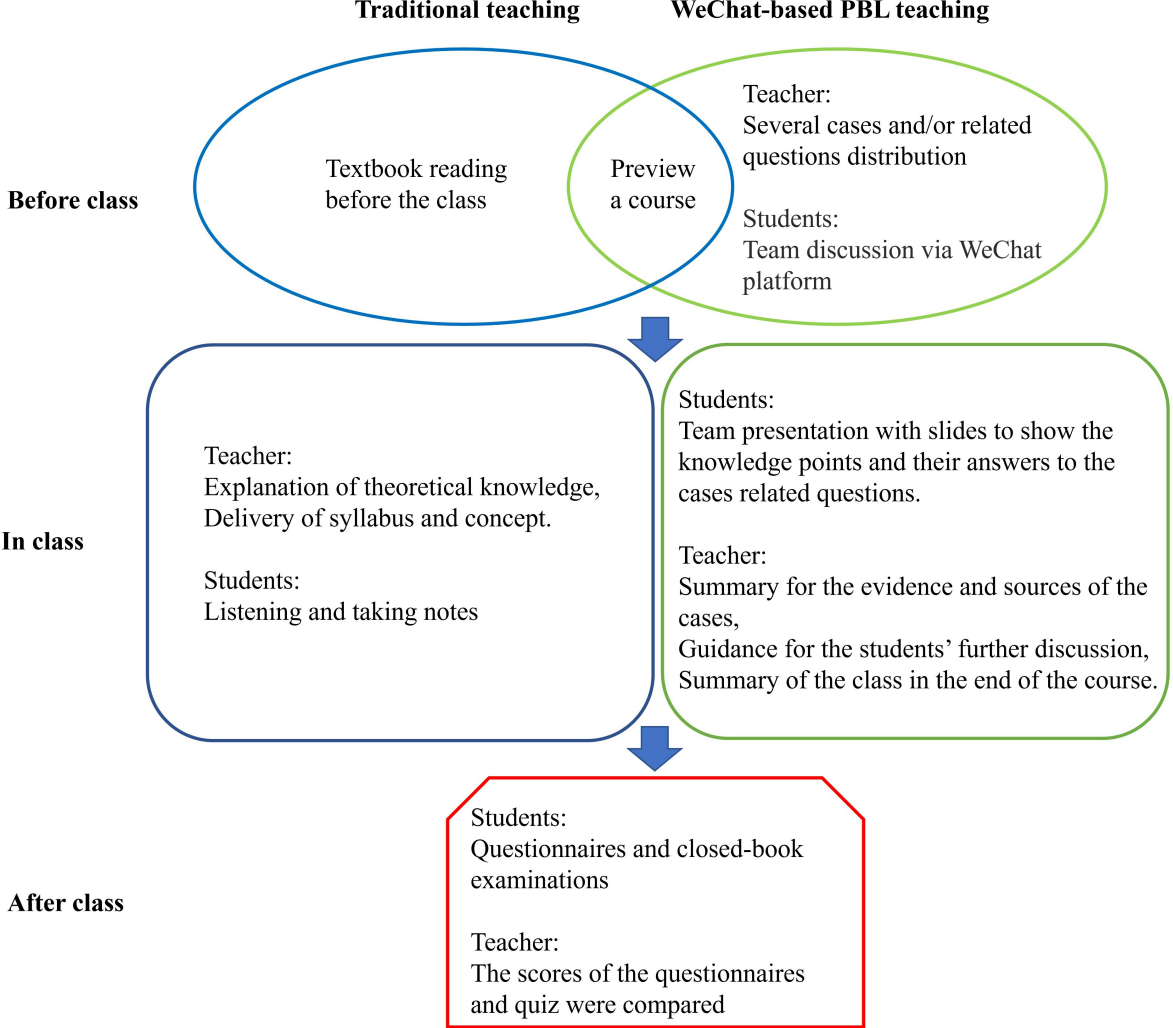
The flowchart shows the 2 different models of teaching and learning in ophthalmology for Chinese medical students. PBL: problem-based learning.

The traditional teaching model involved the instructor predominantly delivering comprehensive explanations of foundational knowledge, adhering to a prescribed multimedia teaching outline. In this model, students were not divided into smaller groups and were not required to give formal presentations during the course. Both groups completed the same quizzes and questionnaires, and the results did not affect course grades or performance. The study design is summarized in Figure 1.

### Teaching Efficacy Evaluation and Statistical Analysis

The efficacy and satisfaction of both teaching methods were evaluated through a combination of questionnaire surveys for subjective assessment and examinations for objective evaluation.

#### Subjective Evaluation

The questionnaire (Table 2) was designed and modified based on the Course Experience Questionnaire by Ramsden [24] and the Study Process Questionnaire by Biggs [25]. All the students completed questionnaires to assess their perceptions and self-evaluated competence at the end of the teaching course.

**Table 2.** Specific questions used in the questionnaire

Topic area	Questions
The course improves my learning motivation	“Can you analyze the impact of the study findings on the field?”
The course helps me to acquire knowledge	“Can you define the main terms used in the study?”
The course is helpful for passing the final examination	“What are your thoughts on the effectiveness of the methods used in the study?”
The course encourages me to express my opinions	“In this course, do you feel that your opinions are respected?” “In this course, do you often take the initiative to express your opinions?”
This course occupies too much of my spare time	“How much time do you spend in WeChat-based problem-based learning by using this method to communicate with teachers and group members?”
The course improves my communication skills	“During discussions, does the instructor give you sufficient time to express your opinions?”
The course improves my self-learning skills	“What are the key concepts discussed in the study material?”
The course improves my clinical thinking ability	“How would you apply the concepts learned to a real-world scenario?”
The course improves my ability to analyze and solve problems	“How much do you agree that this course has provided you with the tools and techniques to effectively solve problems?” “How often have you used the analytical skills you learned in the course to solve real-world problems outside the classroom?

#### Objective Evaluation

Both groups then undertook an identical closed-book examination comprising essay questions, true or false questions, judgment questions, and case analyses to assess students’ memory, comprehension, and clinical application abilities. The examination questions were formulated based on the 9th edition of *Ophthalmology*, an ophthalmology textbook published by the People’s Medical Publishing House [23]. Notably, both the examination format and evaluation criteria were standardized across both groups. To maintain objectivity in scoring the theoretical examination, 3 instructors performed a blinded evaluation of the students’ responses.

Statistical analyses were performed using SPSS (version 11.0; IBM Corp) software. Measurement data were expressed as means (SDs). Questionnaire data were analyzed using the Mann-Whitney *U* test, and examination scores across the two groups were compared using the independent samples 2-tailed *t* test. A *P* value <.05 was considered statistically significant.

## Results

### Participants’ Demographic Data

**Figure 2.**
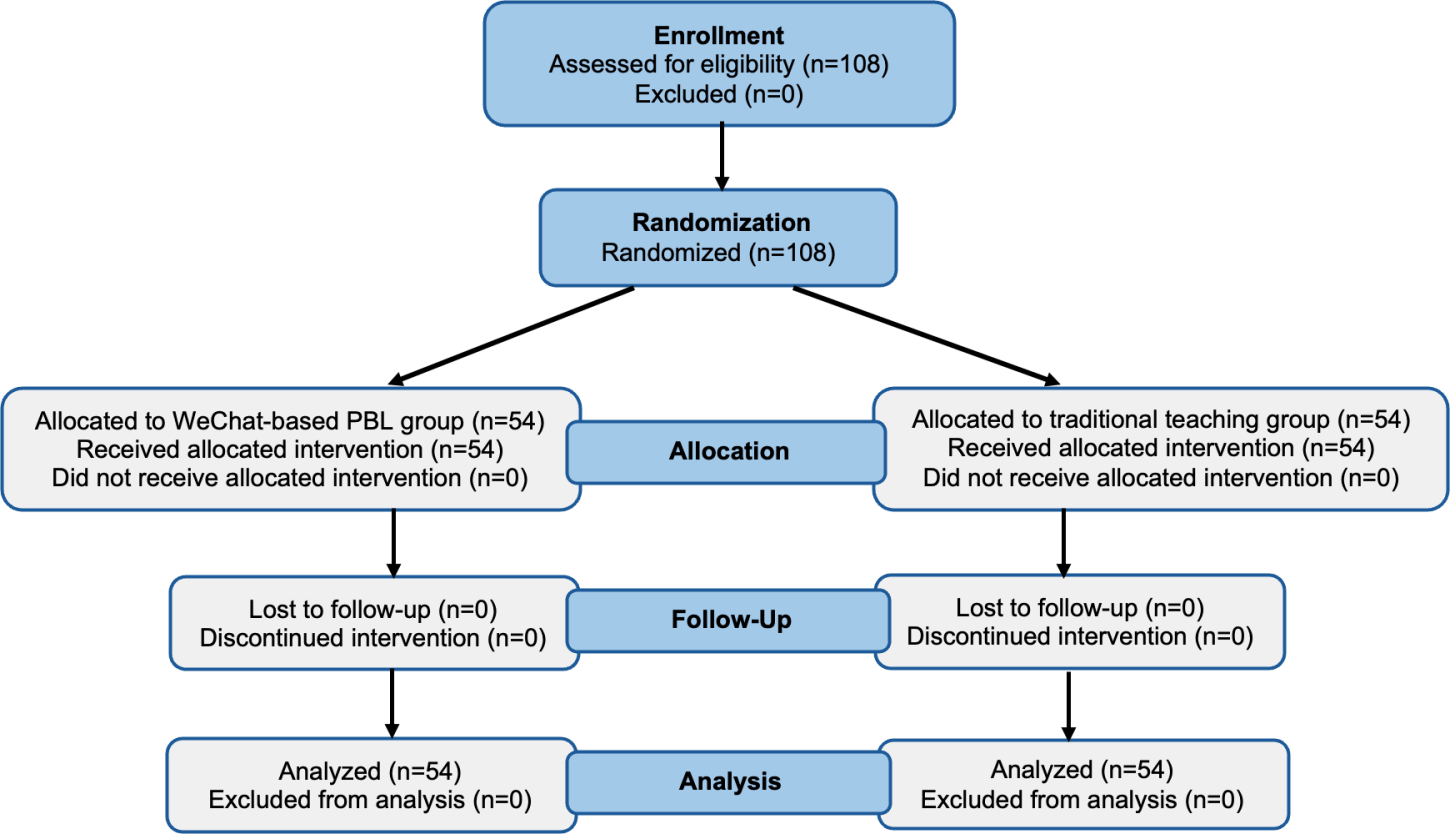
Consolidated Standards of Reporting Trials (CONSORT) flowchart.

Figure 2 shows a flowchart of participation for the 108 medical students in this study. Among them, 54 (50%) students were randomly selected to participate in the WeChat-based PBL, while the other 54 (50%) received traditional teaching. Among the participants, there were 42 (38.9%) male and 66 (61.1%) female individuals who were aged 20 to 22 years. The mean age of the WeChat-based group was 20.426 (SD 0.602) years, and the mean age of the traditional teaching group was 20.296 (SD 0.571) years. There was no significant difference in gender and average age between the two groups (Table 3).

**Table 3.** Participants’ demographic data of the Second Xiangya Medical College of WeChat-based problem-based learning (PBL) and traditional teaching groups.

Characteristics	WeChat-based PBL (n=54)	Traditional teaching (n=54)	*P* value
Age (y), mean (SD)	20.426 (0.602)	20.296 (0.571)	.12^a^
Sex, n (%)			.48^b^
Male	23 (42.6)	22 (40.7)	
Female	31 (57.4)	32 (59.3)	

a For age, independent sample *t*_106_*=*1.188.

b For sex, χ2=1.0.

### Comparison of the WeChat-Based PBL Method and Traditional Teaching Method

The closed-book examination was administered 1 week after course completion to allow students sufficient time for review while minimizing long-term memory effects. This clarification has been included in the revised manuscript. Examination questions were designed by 3 instructors and included 6 types of questions: single-choice questions, multiple-choice questions, noun definitions, short-answer questions, essay questions, and comprehensive questions. The exam assessed 3 cognitive levels—memorization, understanding, and application, which accounted for 50%, 20%, and 30% of the total score, respectively. The memorization section evaluated students’ ability to recall fundamental facts, definitions, and terminology. The understanding section assessed their capacity to interpret information, explain concepts in their own words, and demonstrate deeper comprehension beyond simple recall. The application section examined students’ ability to apply their knowledge and understanding to new or practical situations, such as problem-solving, formula application, and case analysis. Notably, the sections on understanding and application emphasized students’ flexible mastery of knowledge and its practical implementation. The goal was to enable students to apply theoretical knowledge in clinical contexts, thereby improving their diagnostic and treatment skills as well as their ability to analyze and solve clinical problems.

Our analysis indicated that although students in the WeChat-based PBL group scored marginally lower in memorization compared to their peers in the traditional teaching group (traditional vs WeChat groups: 37.6, SD 2.8 vs 32.0, SD 4.1; *t*_106_=-8.426; *P*=.006; n=54), they exhibited markedly superior levels of understanding (traditional vs WeChat groups: 24.1, SD 1.8 vs 28.0, SD 1.3; *t*_106_=12.753; *P*=.007; n=54) and knowledge application (traditional vs WeChat groups: 24.3, SD 1.9 vs 27.6, SD 1.3; *t*_106_=10.899; *P*=.008; n=54). However, when considering the total score, there was no significant difference between the two groups (traditional vs WeChat: 85.8, SD 4.9 vs 87.4, SD 5.1; *t*_106_=1.694; *P*=.60; n=54).

All 108 (100%) students responded to the questionnaire for the comparison of students’ perceptions between the WeChat-based PBL and lecture-based class (Table 4). More students believed (rather than did not believe) that the WeChat-based PBL class enhanced their learning motivation (traditional group: mean 3.5471, SD 0.7915; WeChat group: mean 1.8333, SD 0.5746; *P*=.004), helped them acquire knowledge (traditional group: mean 3.6667, SD 0.7770; WeChat group: mean 1.8704, SD 0.7017; *P*<.001), and improved their performance on the final examination (*P*=.001). They also believed that it encouraged them to express opinions (traditional group: mean 3.5556, SD 0.7181; WeChat group: mean 1.9630, SD 0.5482; *P*=.001), enhanced their communication skills (traditional group: mean 3.8889, SD 0.8393; WeChat group: mean 1.7222, SD 0.5961; *P*<.001), improved their self-learning ability (traditional group: mean 3.5741, SD 0.7673; WeChat group: mean 1.8519, SD 0.4917; *P*<.001), improved their clinical thinking (traditional group: mean 2.9444, SD 0.8777; WeChat group: mean 1.9630, SD 0.6132; *P*=.012), and enhanced their ability to analyze and resolve problems (traditional group: mean 3.2037, SD 0.6553; WeChat group: mean 1.8519, SD 0.5287; *P*=.001). However, a majority of students in the WeChat-based PBL class felt that the course occupied too much of their spare time (traditional group: mean 3.7963, SD 0.7618; WeChat group: mean 1.9630, SD 0.5482; *P*<.001).

**Table 4.** Comparison of students’ perceptions and self-evaluation between WeChat-based problem-based learning (PBL) method and traditional teaching method. The results were ranked as a Likert scale from 1 to 5 (1=strongly agree, 2=agree, 3=neutral, 4=disagree, and 5=strongly disagree).

Questions	WeChat-based PBL, mean (SD)	Control (traditional teaching), mean (SD)	Mann-Whitney *U* test	*P* value	Effect size
The course improves my learning motivation	1.8333 (0.5746)	3.5741 (0.7915)	1012	.004	2.5169
The course helps me to acquire the knowledge	1.8704 (0.7017)	3.6667 (0.7770)	914	<.001	2.4264
The course is helpful for passing the final examination	2.7593 (0.7507)	3.2593 (0.8284)	975.5	<.001	0.6325
The course encourages me to express my opinions	1.9630 (0.5482)	3.5556 (0.7181)	963	.001	2.4930
This course occupies too much of my spare time	3.3148 (0.8201)	3.7963 (0.7618)	714	<.001	0.6083
The course improves my communication skills	1.7222 (0.5961)	3.8889 (0.8393)	725	<.001	2.9765
The course improves my self-learning skills	1.8519 (0.4917)	3.5741 (0.7673)	930.5	<.001	2.6725
The course improves my clinical thinking ability	1.9630 (0.6132)	2.9444 (0.8777)	1072	.012	1.2964
The course improves my ability to analyze and solve problems	1.8519 (0.5287)	3.2037 (0.6553)	932	.001	2.2706

## Discussion

### Principal Findings

This study aimed to assess the effectiveness of WeChat-based PBL in ophthalmic education compared with traditional teaching methods. Our analysis revealed that while students in the WeChat-based PBL group exhibited slightly lower scores in memorization than those in the traditional teaching group, they demonstrated significantly higher levels of understanding and application of knowledge. This suggests that the WeChat-based PBL approach fosters deeper engagement, allowing students to grasp critical concepts more effectively, despite having less focus on rote memorization. Moreover, students in the WeChat group reported enhanced collaboration, motivation, and willingness to participate actively in their learning process.

WeChat has been used as a platform for PBL because of its time efficiency and convenience [26-28]. Students were able to present questions and engage in discussions with instructors at any time and from any location via WeChat. Moreover, it allows the sharing of images, videos, links, and other related resources. This capability greatly aids discussion and ensures that all members can access case-related or problem-related resources promptly. Everyone can share their opinions and suggestions, receiving feedback from others anytime and anywhere. This timely and efficient communication via WeChat offers a better understanding among team members [26,27,29]. Successful collaboration guarantees the productivity of the team, and instructors can actively participate in students’ discussions and debates, responding to questions and offering advice to enhance the efficiency of the learning process [26,27].

Although the primary benefit of WeChat-based PBL is time-saving and convenience, as traditional PBL can be a time-intensive approach [30], the perception that WeChat-based PBL occupied too much spare time underscores the need for careful consideration of time management and workload in educational program design. One of the reasons could be the additional time dedicated to self-learning [31] and discussion among the team members increased the pressure and burden on the students. Increased preparation time can lead to deeper understanding and better retention of the material as students engage with content more thoroughly before class discussions. This proactive approach enhances participation during PBL sessions and ultimately improves learning outcomes. To optimize learning efficiency while preventing overload, several refinements are warranted. Specifically, preclass materials should be streamlined to emphasize key concepts and reduce redundancy, and brief in-class reviews can help reinforce complex topics, minimizing postclass study demands. Periodic guided learning sessions can also be scheduled to help balance students’ independent study and supervised learning time. These refinements aim to alleviate cognitive load while maintaining the effectiveness of the teaching model. Moreover, it is essential to refine the new teaching approach to effectively integrate the advantages of the PBL model with the interactive capabilities of the WeChat platform. Furthermore, instructors should also receive appropriate training to embrace this innovative teaching approach [32]. Dolmans and Wolfhagen [33] have highlighted that a tutor’s actions significantly influence both the productivity and effectiveness of a PBL group’s efforts. To enhance the effectiveness of tutors in PBL tutorials, ongoing training that encourages reflective practice on their development as educators is recommended [34,35]. These efforts can effectively strike a balance between optimal learning outcomes and manageable student workload.

At the same time, the increase in students’ out-of-class learning hours also implied additional workload for instructors. Although instructor workload in asynchronous chat facilitation was not quantitatively measured, informal feedback indicated a moderate increase. Instructors reported spending an additional 15 to 20 minutes per day monitoring discussions and providing timely feedback. Despite this, they generally considered this extra time acceptable and valuable, as it enabled closer observation of students’ reasoning and early identification of misconceptions. The main challenges involved balancing prompt responses with other duties and managing message overload during peak activity. Nonetheless, most instructors agreed that the improved engagement and learning outcomes justified the additional effort. A rotating instructor schedule for online facilitation and setting clearer expectations for response timelines may help balance instructional workload while maintaining effective student-teacher interaction.

Our statistical analysis revealed that the WeChat-based PBL group exhibited slightly lower scores in memorization than the traditional teaching group. However, their levels of understanding and application of knowledge were higher than the traditional teaching group. This discrepancy may be attributed to the engagement of students in the WeChat-based PBL group with a broader array of relevant information, extending beyond textbook content. Consequently, while the WeChat-based PBL group demonstrated a better performance in understanding and application of knowledge, their memorization of textbook-specific information was slightly weaker. Nevertheless, the WeChat-based PBL group showed advantages in mastering key knowledge points and achieving higher total scores.

However, several factors must be considered while implementing WeChat-based PBL. For instance, it is advisable to keep the number of students in each team small, as smaller groups facilitate easier administration and instruction. Teachers should contact students at least 1 week in advance to provide questions or case reports, ensuring both teachers and students are well prepared to achieve optimal educational outcomes. By adapting to real-time situations, teachers can enhance students’ interest in learning, invigorate the classroom dynamics, and correct students’ misconceptions. Teachers should act as a resource and facilitator, responding promptly to students’ inquiries throughout their research. Overall, the WeChat-based PBL model marks a significant departure from the traditional didactic “cramming education” approach, fostering a more engaging and active learning environment. Therefore, WeChat-based PBL should be increasingly incorporated into future ophthalmology education to enable continuous identification and refinement of relevant issues [36].

### Limitations

There are several limitations that should be acknowledged. First, while students covered the same core content on PACG, differences in teaching methodologies could have influenced memorization outcomes and overall comprehension. This research was performed targeting a single textbook chapter on PACG. Therefore, the conclusion requires further verification in other subject fields. Second, questionnaire results indicated that students in the WeChat-based PBL group spent more extracurricular time preparing course materials. Therefore, understanding their perceptions and attitudes toward this additional workload is particularly important. However, as the students involved in this study have already graduated, it was not possible to collect further feedback from them. This limitation prevents a more comprehensive understanding of their long-term perceptions of the learning burden and its potential impact on sustained engagement and clinical competency development. Future research will include longitudinal follow-up surveys or interviews to further evaluate students’ attitudes, learning experiences, and workload perceptions. Third, the necessity for student participation in additional discussions and presentations may have affected their independent study time and overall academic performance. Fourth, the lack of blinding may have introduced biases, as awareness of the teaching method could have influenced both students’ and instructors’ expectations and engagement levels. Fifth, the generalizability of our findings is also limited by the single-institution context and the sample size of 108 fifth-year undergraduate medical students, which may not reflect broader educational settings or diverse student populations. Finally, in our study, we did not compare the effects of traditional PBL and WeChat-based PBL methods. Further research should compare the functions between these two approaches in future studies. Moreover, future research should explore the long-term impacts of WeChat-based PBL on learning outcomes across different medical disciplines and institutions. Further empirical testing is necessary to assess the effectiveness of this approach in various contexts and to identify best practices for implementation. Optimizing digital communication tools such as WeChat holds great promise for enhancing educational experience and fostering collaborative learning in medical education.

### Conclusions

In summary, compared to traditional teaching, integrating PBL methods combined with WeChat as a communication platform improved the effectiveness of ophthalmic education. Although students invested more time in preclass, they did not perceive it as a waste of their spare time. Instead, they recognized that the WeChat-based PBL mode facilitated more effective communication among students and between students and teachers. It enhanced their motivation to learn; promoted knowledge acquisition; encouraged expression of opinions; and improved self-learning abilities, clinical reasoning, and problem-solving. This innovative teaching mode may represent a superior alternative to conventional teaching methods. However, further exploration to optimize the students’ spare time for effective online communication is warranted. This new method merits continued refinement and evaluation based on the findings of this study.

## Abbreviations

PACG: primary angle closure glaucoma
PBL: problem-based learning
